# Current changes in the epidemiology of fall-related injuries in Al Ain City, United Arab Emirates

**DOI:** 10.1371/journal.pone.0257398

**Published:** 2021-09-16

**Authors:** Arif Alper Cevik, David O. Alao, Hani O. Eid, Michal Grivna, Fikri M. Abu-Zidan

**Affiliations:** 1 Department of Internal Medicine, College of Medicine and Health Sciences, UAE University, Al-Ain, Ain, United Arab Emirates; 2 Department of Emergency Medicine, Al Ain Hospital, Al-Ain, United Arab Emirates; 3 Emergency Department, Mediclinic Airport Road Hospital, Abu Dhabi, United Arab Emirates; 4 Institute of Public Health, College of Medicine and Health Sciences, UAE University, Al-Ain, Ain, United Arab Emirates; 5 Department of Surgery, College of Medicine and Health Sciences, UAE University, Al-Ain, Ain, United Arab Emirates; John Hunter Hospital and University of Newcastle, AUSTRALIA

## Abstract

**Background:**

Falls in the Gulf countries are the second most common cause of injuries. The United Arab Emirates government implemented various preventive measures to decrease injuries in the country. We aimed to evaluate the changes in the epidemiology of fall-related injuries in Al-Ain City over the last decade.

**Methods:**

Data of hospitalized patients who presented with fall-related injuries to the Al-Ain Hospital during the two periods of March 2003 to March 2006 and January 2014 to December 2017 were compared. This included patients’ demographics, mechanism, location, anatomical distribution and parameters related to injury severity. Non-parametric tests were used for the statistical analysis.

**Results:**

882 in the first and 1358 patients in the second period were studied. The incidence of falls decreased by 30.5% over ten years. The number of elderly, female patients, and UAE nationals increased, (*p* < 0.001, *p* = 0.004, and *p* < 0.001). Falls from height decreased by 32.5% (*p* < 0.001) while fall on the same level increased by 22.5% (*p* < 0.001). Fall-related injuries at home have increased significantly by 22.6% (*p* <0.001), while falls in workplaces decreased by 24.4% (*p* <0.001).

**Conclusions:**

Our study showed that the overall incidence of falls decreased compared to a decade ago. The preventive measures were effective in reducing falls from height and workplace injuries. Future preventive measures should target falls at the same level and homes.

## Introduction

Fall-related injuries are one of the leading causes of morbidity and mortality worldwide. Its global mortality increased from 10.2% in 2000 to 13.5% in 2016 [1, 2]. Falls are more common in young males under the age of 44 years compared with older adults. However, the majority of fall-related deaths occur in the elderly population [1, 2]. Although the preponderance of injuries is in the low- and middle-income countries [3], falls are the third most common cause of injury-related deaths in high-income countries [4].

Falls are the second common cause of injury in the Gulf countries [5]. Construction work is the main contributor to falls from height in these countries [5–8]. Like reports from Qatar, Iraq, Oman, and Saudi Arabia falls are a significant cause of injuries in the United Arab Emirates (UAE) [8–13].

Although the Disability Adjusted Life Years (DALYs) for fall-related injuries decreased by 20.8% from 1990 to 2013 globally [14], this varied in different countries. The Health Authority of Abu Dhabi (HAAD) estimated the annual incidence of occupational injury in the UAE in 2007 to be 136/100,000 workers. Over half of them were due to falls, mostly from height. More than two-thirds of the fall from height occurred in labourers of whom 99% were males from the Asia Subcontinent [15]. The Health Authority of Abu Dhabi, therefore, listed occupational fatality as one of its top ten public health priorities [16]. Several legislative measures were taken to reduce the high incidence of fall-related injuries.

The United Arab Emirates Labour Act, Federal Law 8 (1980) and its amendments (1982) regulates and promote workplace safety and affirm the enterprise’s obligation towards protecting employees’ health and safety [17]. This includes an obligation for employers to provide protective personal equipment for their employees including body slings, helmets, gloves and boots in the workplace [17]. The Environmental, Health and Safety Management System was established in 2009 and in February 2010, the Occupational Health and Safety Centre of Abu Dhabi (OSHAD) was established to ensure the enforcement of occupational safety in the workplace [18]. The management framework of the centre integrates occupational safety as an important component of the business system [18]. Failure to comply with health and safety regulations have serious consequences for individuals and employers. Violations of this law could attract a penalty of imprisonment and or a fine of up to 50,000 AED [19]. In addition, the child jockey law of 2005 prohibits the use of children as jockey at camel races and recommend robots instead. This has resulted in a significant reduction in falls from camels [20]. These measures may have changed the severity and pattern of fall-related injuries in the country.

We aimed to evaluate the changes in the epidemiology of fall-related injuries in Al-Ain City that occurred over the last decade.

## Materials and methods

### Ethical approval and consent to participate

The Human Research Ethics Committee of Al-Ain Hospital, Al Ain, United Arab Emirates has given Ethical approval for this study (AAHEC-03-20-008). The patients or their caregivers signed a written informed consent to use the patients’ data in research. Patient’s identifiers were protected, and data were fully anonymous during the analysis.

### Study setting and population

Al-Ain Hospital is the main trauma centre in our city and treats 80% of Al-Ain city’s trauma cases. Data of patients who presented with fall-related injuries to Al-Ain Hospital and were admitted for more than 24 hours or died after arrival to the hospital during the two periods of March 2003 to March 2006 and January 2014 to December 2017 were extracted from the Al-Ain Hospital Trauma Registry. The population of the Al-Ain City were estimated to be 460 000 during the first period [21] and 766,009 during the second period [22].

Data collection in the first trauma registry was from 2003 to 2006 [23, 24]. It was a research feasibility study funded by the UAE University. The second official registry was established in 2013 by the Department of Health, Abu Dhabi Emirate as part of the trauma quality assurance program of Al-Ain Hospital [25]. Data were prospectively collected in both registries; by trauma fellows in the first period and by registry nurses in the second period. The first registry was developed by the Trauma Group, UAE University, while the second registry was adopted after the American College of Surgeons Trauma Registry. Data were validated regularly to assure its quality. The registry of the first period was of a high standard developed by international experts in this area. The full-time research fellows who collected the data in the first period were directly trained and supervised by the senior author (FAZ), who has extensive experience in developing trauma registries and research training. Variables that were similar in both registries were used for comparison of the two periods.

### Studied variables

Age, gender, nationality (Emirati and non-Emirati), mechanism and anatomical location of the injury, physiological parameters (systolic blood pressure, heart rate), injury severity score (ISS), new injury severity score (NISS), Glasgow Coma Scale (GCS), intensive care unit (ICU) admission, length of hospital stay), and mortality was extracted from the trauma registry.

### Statistical analysis

Al-Ain Hospital takes care of around 80% of the total hospitalized trauma patients in our city. Accordingly, the overall admitted fall-related injuries in Al-Ain city is estimated to be 100/80 (1.25) of those admitted to Al-Ain Hospital. The annual incidence of fall-related injuries during the two studied periods was calculated as follows: (1.25 × annual admissions) / (population/100000). Categorical data were presented as number (percentage), ordinal data as median (range), and continuous data as mean (standard deviation). To compare categorical data of two independent groups, Pearson’s Chi-square or Fisher’s Exact test were used as appropriate. To compare continuous or ordinal data, the Mann-Whitney U test was used. Bonferroni correction was used to protect against the increased risk of type I error by defining the accepted p-value after subgroup analysis to be 0.05 divided by the number of subgroup comparisons. It was 0.025 in the current paper [26]. Statistical Package for the Social Sciences (IBM-SPSS version 26, Chicago, Il) was used for all analyses.

## Results

There were 2573 and 3519 trauma patients in the registry during the first and second periods, respectively. Eight hundred and eighty-two (34.3%) patients in the first and 1358 (38.6%) patients in the second period had fall-related injuries. The mechanism included falls from height or fall on the same level. An average of 294 patients with fall injury was admitted to the hospital annually during the first period, resulting in an estimated 79.9 per 100.000 population. During the second period, an annual average of 340 patients with fall injuries was admitted to the hospital. The estimated annual incidence for the second period was 55.5 per 100.000 population. The incidence of falls in Al-Ain City decreased by 30.5%.

Patients’ demographics, the mechanism (fall from same level and fall from height), and parameters related to the severity of injury during the two periods are shown in **Table 1**. The patients’ age significantly increased over time (*p* = 0.018). Fall-related injuries in the elderly population also increased by 6.5% (p < 0.001). There was a significant difference between genders. Female patients’ fall-related injuries increased by 5% (p = 0.004). There is also a significant increase in UAE nationals’ fall-related injuries (*p* < 0.001).

**Table 1:** Patients’ demographics, mechanism and parameters during the first period (2003–2006) and second period (2014–2017).

Variable	Years 2003–2006	Years 2014–2017	*p-*value
	(n = 882)	(n = 1358)	
**Age** *	33 (0.5–100) 32.6 (17.4)	33 (1–105) 36.3 (21.1)	0.018
Age categories			<0.001
Age ≥65	43 (4.9%)	155 (11.4%)	
Age <65	839 (95.1%)	1203 (88.6)	
**Gender**			0.004
Male	727 (82.4%)	1051 (77.4%)	
Female	155 (17.6%)	307 (22.6%)	
**Nationality**			<0.001
UAE	129 (14.8%)	302 (22.4%)	
Non-UAE	745 (85.2%)	1048 (77.6%)	
**Mechanism**			<0.001
Fall from same level	409 (46.4%)	936 (68.9%)	
Fall From Height	473 (53.6%)	422 (21.1%)	
**Methods of arrival**			<0.001
Ambulance	70 (7.9%)	332 (30%)	
Other	812 (92.1%)	773 (70%)	
**Physiologic Parameters**			
SBP	129 (74–232)	134 (21–265)	<0.001
Heart rate	87 (50–188)	86 (18–171)	0.142
GCS	15 (3–15)	15 (3–15)	0.189
ISS*	4 (1–34), 5.0 (4.15)	4 (1–36), 6.7 (4.97)	<0.001
NISS*	4 (1–48), 6.1 (5.72)	4 (1–59), 8.6 (6.63)	<0.001
**Outcome Variables**			
ICU admission	30 (3.4%)	53 (3.9%)	0.537
Hospital stay	5 (1–150)	4 (1–121)	<0.001
Death	2 (0.2%)	5 (0.4%)	0.549

Data are presented as median (range) or number (%) as appropriate. The numbers may not add up to the total number of patients because of missing data. Percentages are those from the available data.

* Mean (standard deviation) is presented for statistically significant ordinal data because the median is the same.

*p*-value = Fisher’s Exact test for categorical data and Mann Whitney U test for ordinal or continuous data.

There is a significant difference in the mechanism of falls between the two periods. Falls from heights decreased by 32.5% and falls from the same level increased by 22.5% (*p* < 0.001). There was a significant increase of the systolic blood pressure in the second period compared with the first period (p <0.001), median (range) 129 (74–232) mmHg compared with 134 (21–265) mmHg, respectively. However, the median (range) of heart rates were similar; 87 (50–188) compared with 86 (18–171) beats per minute respectively. Likewise, there was no difference in the GCS over the two periods: 15 (3–15) compared with 15 (3–15) respectively. Both ISS and NISS were higher in the second period (*p* < 0.001). The patients who were admitted in the second period had shorter hospital stay compared with the first period. The median (range) of hospital length of stay was 5 (1–150) compared with 4 (1–121) (*p* < 0.001). There was no difference in mortality between the two periods.

There is a significant difference in the locations of fall-related injuries between the two periods (**Table 2**). Fall-related injuries at home and public areas increased by 22.6% (*p* <0.001) and 7.6% (*p* <0.001), respectively. However, falls in workplaces, farms, and off-roads decreased by 24.4% (*p* <0.001), 1% (*p* = 0.010), and 4.3% (*p* <0.001), respectively.

**Table 2:** Location of injuries during the first (2003–2006) and second period (2014–2017).

Location	Years 2003–2006	Years 2014–2017	*p-*value
	(n = 882)	(n = 1337)	
	Number (%)	Number (%)	
**Home**	324 (36.7)	793 (59.3)	<0.001
**Street/Highway**	12 (1.4)	21 (1.6)	0.687
**Workplace**	435 (49.3)	333 (24.9)	<0.001
**Farm**	16 (1.8)	10 (0.7)	0.010
**Off-road**	56 (6.3)	28 (2.1)	<0.001
**Public area**	20 (2.3)	132 (9.9)	<0.001
**Others**	19 (2.2)	20 (1.5)	0.481

*p*-value (Fisher’s Exact test).

There is a significant increase in abdominal injuries in the second period (*p* = 0.041), while chest injuries decreased significantly (*p* = 0.037). Head, face, and neck, spine, upper and lower extremities did not show any difference (**Table 3**).

**Table 3:** Anatomical distribution of fall-related injuries during the first (2003–2006) and second period (2014–2017).

	Years 2003–2006	Years 2014–2017	
	(n = 1041 regions)	(n = 1860 regions)	
Region	Number (%)	Number (%)	*p-*value
**Head, face and neck**	157 (15.1)	327 (17.6)	0.081
**Chest**	113 (10.9)	158 (8.5)	0.037
**Abdomen**	34 (3.3)	90 (4.8)	0.041
**Spine**	115 (11.1)	237 (12.7)	0.177
**Upper extremity**	260 (24.9)	437 (23.5)	0.371
**Lower extremity**	362 (34.8)	611 (32.8)	0.292

*p*-value (Fisher’s Exact test).

**Table 4** shows the sub-group analysis of patients by the mechanism. In the falls from the same level category, the patients’ age (*p* <0.001), number of elderly patients (*p* = 0.002), arrivals by ambulance (*p* < 0.001), patients’ SBP (*p* = 0.011), ISS (*p*<0.001) and NISS (*p* <0.001) increased in the second period. In the same category, patients’ heart rate and hospital length of stay were significantly decreased in the second period. Arrival by ambulance of patients who fell from height significantly increased by 30.3% (*p*<0.001) in the second period. The patient’s ISS and NISS were also higher in the second period (*p* <0.001 for both), while the hospital length of stay was shorter (*p* = 0.003).

**Table 4:** Comparison of the mechanism of fall-related injuries during the first (2003–2006) and second period (2014–2017).

	Falls from Same Level			Falls from Height		
	n = 1345			n = 895		
	2004–2007	20014–2017		2004–2007	20014–2017	
	n = 409	n = 936		n = 473	n = 422	
	Median (range)	Median (range)	*p*-value	Median (range)	Median (range)	*p*-value
**Age**	30 (0.5–100)	33 (1–105)	<0.001	35 (1.8–65)	33 (2–78)	0.786
**Age categories**			0.002			0.999
Age ≥65	41 (10%)	154 (16.5)		2 (0.4%)	1 (0.2%)	
Age <65	368 (90%)	782 (83.5)		471 (99.6)	421 (99.8%)	
**Gender**			0.999			0.574
Male	285 (69.7%)	652 (69.7%)		442 (93.4%)	399 (94.5%)	
Female	124 (30.3%)	284 (30.3%)		31 (6.6%)	23 (5.5%)	
**Nationality**			0.166			0.478
UAE	104 (25.6%)	274 (29.4%)		25 (5.3%)	28 (6.7%)	
Non-UAE	302 (74.4%)	657 (70.6%)		443 (94.7%)	391 (93.3%)	
**Methods of arrival**			<0.001			<0.001
Ambulance	11(2.7%)	188 (24.4%)		59 (12.5%)	144 (42.8%)	
Other	398 (97.3%)	581 (75.6%)		414 (87.5%)	192 (57.1%)	
**Physiologic Parameters**						
SBP	128 (90–232)	134 (21–265)	0.011	129 (74–222)	133 (60–234)	<0.001
Heart rate	90 (59–188)	86 (18–171)	0.004	85 (50–167)	84 (51–157)	0.499
GCS	15 (3–15)	15 (8–15)	0.656	15 (3–15)	15 (3–15)	0.598
ISS*	4 (1–25)	4 (1–29)	<0.001	4 (1–34)	8 (1–36)	<0.001
	4.73 (2.89)	5.72 (3.69)				
NISS	4 (1–32)	8 (1–51)	<0.001	4 (1–48)	9 (1–59)	<0.001
**Outcome Variables**						
ICU admission	3 (0.7%)	18 (1.9%)	0.316	29 (6.1%)	35 (8.3%)	0.210
Hospital stay	4 (1–150)	3 (2–91)	<0.001	6 (1–93)	5 (1–121)	0.003
Death	1 (0.2%)	1 (0.1%)	0.563	1 (0.2%)	4 (0.9%)	0.129

Data are presented median (range) or number (%) as appropriate. The numbers may not add up to the total number of patients because of missing data. Percentages are those from the available data.

* Mean (standard deviation) is presented for statistically significant ordinal data because the median is the same.

*p*-value = Fisher’s Exact test for categorical data and Mann Whitney U test for ordinal or continuous data.

## Discussion

Our study has shown that the incidence of fall-related injuries decreased in our setting. Fall-related injuries increased in the elderly, female population, and UAE nationals. Although falls from the same level, in-homes and public places increased, falls from height and workplaces decreased. The severity of injuries was higher, but hospital length of stay decreased over time. The mortality did not change.

Some countries in our region, such as Iraq, have reported an increased incidence of accidental falls [10]. In contrast, fall-related injuries reduced overtime in our city, which is similar to the global decrease [14]. However, the Gulf region countries showed the lowest decrease (≤10%) in the incidence of fall-related injuries and shared this category with South Asian and African countries [14]. One of the significant determinants of fall-related injuries is its location. Workplace-related falls from height are still common in our setting because of the construction demands [27]. We think that the decrease in fall from height in the current study reflects the successful implementation of safety regulations at the workplace in our setting [28].

Fall-related injuries in our study were mainly in males. In contrast, other studies showed higher incidence in females [29, 30]. This can be attributed to the high percentage of male expatriate manual workers in the construction industry in our city. Nevertheless, and like others, fall-related injuries in females, which are mainly on the same level, has increased in our study [31, 32].

Geriatric falls is a serious public health problem [33]. Living alone, the effect of medications, movement disorders, weak sensory power, reduced mobility, and arthritis are risk factors for falls in the elderly population, even if physically active [34–36]. UAE has a fast-ageing population compared with the other Gulf countries [37]. Although injury prevention and developments in the trauma system in our city reduced injuries by 38.2% and mortality by 56% in hospitalized trauma patients [25], our study has shown that geriatric falls increased over time.

Despite the increased severity of injuries of hospitalized fall-related patients in the second period, hospital stay and mortality were less. This can be attributed to developments in the trauma system, which includes better pre-hospital field medical management, faster transfer to the hospital and better in-hospital trauma care, including the emergency department, surgical interventions, and critical care.

Fall prevention programs should be implemented appropriately [8, 38]. Despite the significant decrease in work-related fall-related injuries and fall from height in the current study, there is an urgent need to reduce falls in public areas and homes.

### Limitations

There are several limitations to our study. *First*, the data studied were from a single hospital in our city which cannot be generalized to the whole country. Nevertheless, we think that observing the preventive interventions and evaluating their effects gives a strong example to follow within our region. *Second*, we studied injured patients who were hospitalized for more than 24 hours or who died in the hospital. Therefore, this group does not represent the whole fall-related injuries in our city as it excludes those who did not present to the hospital. *Third*, it would have been better to have a continuous data registry through those years without interruption. This occurred because of financial restraints, which is a common problem facing trauma registries. *Fourth*, we do not have a detailed description of the injury incidents and their contributing factors. These details can give us an in-depth understanding of the possible prevention strategies and should be included in future studies. Finally, we acknowledge that our study may not directly prove the cause-effect relationship of our results and other unstudied factors may have contributed to these effects. Nevertheless, we think that the highlighted legislative and enforcement actions taken in our community to reduce fall from height at workplaces may explain our findings.

## Conclusions

Our study has shown that the overall incidence of falls decreased over time. This was mainly in fall from height at workplaces. Falls at the same level, at home and in the geriatrics increased, indicating that future preventive measures should target falls at the same level at homes.

## Supporting information

S1 Data (XLSX)

## List of abbreviations

GCS: Glasgow Coma Scale
ICU: Intensive Care Unit
ISS: Injury Severity Score
NISS: New Injury Severity Score
UAE: United Arab Emirates
